# Potential antiviral peptides targeting the SARS-CoV-2 spike protein

**DOI:** 10.1186/s40360-022-00627-w

**Published:** 2022-12-02

**Authors:** Ibrahim Khater, Aaya Nassar

**Affiliations:** 1 Biophysics Department, Faculty of Science, Cairo University, Giza, Egypt; 2 Department of Clinical Research and Leadership, School of Medicine and Health Sciences, George Washington University, Washington DC, USA

**Keywords:** SARS-CoV-2, Spike protein, COVID-19, Molecular docking, Peptide blocker, ACE2

## Abstract

**Background:**

The coronavirus disease caused by the severe acute respiratory syndrome coronavirus 2 (SARS-CoV-2) infection became an international pandemic and created a public health crisis. The binding of the viral Spike glycoprotein to the human cell receptor angiotensin-converting enzyme 2 (ACE2) initiates viral infection. The development of efficient treatments to combat coronavirus disease is considered essential.

**Methods:**

An in silico approach was employed to design amino acid peptide inhibitor against the receptor-binding domain (RBD) of the SARS-CoV-2 spike protein. The designed inhibitor (SARS-CoV-2 PEP 49) consists of amino acids with the α1 helix and the β4 - β5 sheets of ACE2. The PEP-FOLD3 web tool was used to create the 3D structures of the peptide amino acids. Analyzing the interaction between ACE2 and the RBD of the Spike protein for three protein data bank entries (6M0J, 7C8D, and 7A95) indicated that the interacting amino acids were contained inside two regions of ACE2: the α1 helical protease domain (PD) and the β4 - β5 sheets.

**Results:**

Molecular docking analysis of the designed inhibitor demonstrated that SARS-CoV-2 PEP 49 attaches directly to the ACE2 binding site of the Spike protein with a binding affinity greater than the ACE2, implying that the SARS-CoV-2 PEP 49 model may be useful as a potential RBD binding blocker.

## Introduction

The coronavirus disease (COVID-19) is an ongoing global pandemic caused by the enigmatic severe acute respiratory syndrome coronavirus 2 (SARS-CoV-2). Since the virus was originally detected, multiple mutated variants of the virus have been identified globally, resulting in surges of infection, despite the vaccine administration. As of March 27, 2022, the pandemic had caused more than 480 million cases and 6.12 million deaths, making it one of the deadliest in history.

In the realm of infectious diseases, a pandemic is a worst-case scenario. During the last two decades, several viral epidemics involving the coronavirus family have been reported, including the Severe Acute Respiratory Syndrome Coronavirus (SARS-CoV) found in 2002 and the influenza epidemic of the H1N1 virus in 2009, while the Middle East Respiratory Syndrome Coronavirus (MERS-CoV) was first identified in 2012 [1].

A human coronavirus (HCoV) is a virus that attacks the respiratory system of humans. *Alphacoronaviruses* 229E and NL63, as well as *Betacoronaviruses* OC43, HKU1, SARS, and MERS, have caused previous coronavirus outbreaks [2]. The most dangerous coronavirus strains have been identified as SARS and MERS. SARS-CoV-2 is the seventh coronavirus to infect humans; four of these coronaviruses, 229E, NL63, OC43, and HKU1, cause mild symptoms similar to common cold symptoms, while the other three coronaviruses, SARS-CoV, MERS-CoV, and SARS-CoV-2, cause severe symptoms that can be difficult to treat, resulting in high hospitalization and mortality rates. The SARS-CoV-2 virus is contagious, highly transmittable among humans, and spreads across countries. The genetic sequence of SARS-CoV-2 was found to be 88% similar to SARS [2–5], indicating that SARS-CoV-2 is a *Betacoronavirus* like SARS and MERS [6]. HCoVs are positive-sense, single-stranded RNA viruses with a genome length of about 30,000 base pairs. Spike protein (S), Nucleocapsid protein (N), Membrane protein (M), and Envelope protein (E) are structural proteins, while polymerase protein and protease protein are non-structural proteins found in HCoVs [7]. The Spike protein, which is one of the structural proteins, generates enormous protrusions from the virus’s surface, giving it the appearance of a crown. The Spike protein mediates the virus’s entry into the host cell [8, 9].

The SARS-CoV-2 Spike protein is involved in cell receptor identification and membrane fusion [9]. The Spike protein is made up of two subunits: the S1 receptor-binding domain (RBD), which recognizes and binds to the host receptor angiotensin-converting enzyme 2 (ACE2), and the S2 subunit, which is in charge of facilitating viral cell membrane fusion [10–14]. During viral infection, the Spike protein is split into S1 and S2 subunits, with S1 subunits released during the transition to the post-fusion conformation [15–20]. The RBD of the S1 subunit directly binds to the peptidase domain (PD) of ACE2 [21], whereas the S2 subunit is responsible for membrane fusion. When S1 binds to the host receptor ACE2, another cleavage site on S2 is exposed, and host proteases cleave it, a step that is necessary for viral infection [22, 23].. The SARS-CoV-2 Spike protein may similarly use ACE2 to infect the host cell [21, 24, 25]..

The genomics research examined approximately 4 K SARS-CoV-2 genomes using large-scale, fast whole-genome sequencing in order to better understand viral transmission and evolution [26], (27), [27, 28]. The first virus strains discovered were the B.1.1.7 lineage, which originated from the United Kingdom, P.1 from Brazil, B.1.351 from South Africa, and delta variant B.1.617.2, which was first discovered in India in December 2020 and was substantially more contagious and sparked a worldwide increase in coronavirus cases. A new variant named Omicron B.1.1.529 was first identified in South Africa in November 2021, and at present, there are a number of Omicron sub-lineages, including BA.2, BA.4, and BA.5, and another recombinant detected, made up of BA.1 and BA.2. The main goal of basic research studies is to find powerful SARS-CoV-2 structural and non-structural protein inhibitors. Recent research looked at a few commercially available antiviral treatments to see if they can be repurposed to inhibit the SARS-CoV-2 main protease (Mpro) and papin-like protease (PLpro), using in-silico screening to look for possible SARS-CoV-2 inhibitors by using molecular docking analysis [29, 30]. Because the main protease protein appears to play an essential role in viral replication, inhibiting it may be effective in blocking the initiation of the infection and the replication chain. On the other hand, few approved antiviral treatments demonstrated high binding probabilities as inhibitors of the SARS-CoV-2 papin-like protease. The current work aims to design an in-silico peptide inhibitor of the SARS-CoV-2 Spike protein by first examining the interactions between ACE2 and RBD of the Spike protein for the three protein data bank (PDB) entries: 1) The crystal structure of the SARS-CoV-2 spike receptor-binding domain bound with ACE2 (PDB IDs: 6M0J), 2) The cryo-EM structure of cat ACE2 and SARS-CoV-2 RBD (PDB IDs: 7C8D), and 3) The SARS-CoV-2 Spike Glycoprotein with one ACE2 bound and one RBD upright in a clockwise direction (PDB IDs: 7A95). The peptide models were computationally designed by applying the PEP-FOLD3 web tool to act as the inhibitors of RBD-ACE2 interaction and developed into 49 amino acids.

## Methods

### Structural analysis

Three structures of the SARS-CoV-2, 6M0J, 7C8D, and 7A95, were selected as models to illustrate the interactions between the SARS-CoV-2 RBD of the Spike protein and ACE2. The protein structures were then downloaded from the Protein Data Bank (PDB), available at (https://www.rcsb.org). The interacting energy between RBD and ACE2 was calculated using MM/GBSA on the HawkDock web server [31]. MM/GBSA is employed to predict the binding free energy and decompose the free energy contributions to the binding free energy of a protein-protein complex in per-residue to help analyze the binding structures. Interface residues between RBD and ACE2 were calculated using MM/GBSA for the three protein structures. The interface residues that were repeated in all structures were extracted.

### The novel peptide design

The web-based tool ESPript (https://espript.ibcp.fr/ESPript/ESPript) is a web tool for extracting and rendering a comprehensive analysis of the protein structure in an automated form. The ESPript program renders sequence similarities and secondary structure elements from aligned sequences with numerous options to optimize and enhance their depiction [32]. The amino acid sequence of ACE2 was aligned with the secondary structure of ACE2 of 6M0J using ESPript version 3. The sequence of the secondary structure, which was obtained and used to design the peptide, contains the most relevant and adjacent repeating ACE2 interface residues.

### Physicochemical properties and solubility prediction

Using the online web server ProtParam (http://web.expasy.org/protparam), the computed parameters, including theoretical pI (isoelectric point), aliphatic index, instability index, estimated half-life in mammalian reticulocytes in vitro, extinction coefficient, molecular weight, and grand average of hydropathicity (GRAVY) of candidate peptides, were determined [33, 34]. The Pepcalc program (http://pepcalc.com) was then used to determine the peptide’s estimated solubility in water [35].

### Secondary structure prediction

PSI-blast based secondary structure PREDiction (PSIPRED) is a protein structure analysis approach. Its algorithm is based on artificial neural networks and machine learning. The PSIPRED prediction method (http://bioinf.cs.ucl.ac.uk/psipred) was used to predict the secondary structure of the peptide [36].

### Tertiary structure prediction and validation

PEP-FOLD3 (https://bioserv.rpbs.univ-paris-diderot.fr/services/PEP-FOLD3) is a fast computational framework for de novo free-biased prediction of linear peptides between 5 and 50 amino acids that allows for the creation of native-like conformations of peptides interacting with proteins when the interaction site is known [37, 38]. The PEP-FOLD3 web tool was applied for three-dimensional (3D) structure prediction of the peptide, yielding five different models that were ranked based on free energies, the ERRAT score [39] and the PROVE pass test [40]. The best model was verified with the PROCHECK [41] program and ProSA-web (https://prosa.services.came.sbg.ac.at/prosa.php) [42].

### Molecular docking

The protein docking server ClusPro 2.0 was used to dock the peptide to the RBD of the Spike protein [43–45]. The ClusPro server (https://cluspro.org) is a widely used tool for protein-protein docking. Ten docking complexes were created. The complexes were ranked according to MM/GBSA interaction energy. After that, the docking complex with the highest interaction energy was chosen.

### Molecular dynamics simulations

The CHARMM-GUI was used to build the protein topologies and parameter files [46–48]. The software package GROMACS-2019 [49], as well as the CHARMM36 force field [50], were carried out for the molecular dynamics (MD) simulation. The system was solvated with TIP3P water in the “add solvation box” [51], and the whole complexes were neutralized by using the Monte-Carlo ion-placing method to add appropriate amounts of K+ and Cl ions. The system was energy-minimized for 5000 steps using the steepest descent algorithm before simulations [52] and equilibrated for 125 ps at a constant number of molecules, volume, and temperature (NVT). Finally, the MD simulations were performed for 100 ns at a constant temperature (310 K), pressure (1 atm), and the number of molecules (NPT ensemble). The number of hydrogen bonds, the radius of gyration (Rg), and the root mean square deviation (RMSD) of the protein atom backbone were displayed as a function of time [53]. The average root mean square fluctuation (RMSF) was then graphed as a function of the residue number.

## Results

**Fig. 1:**
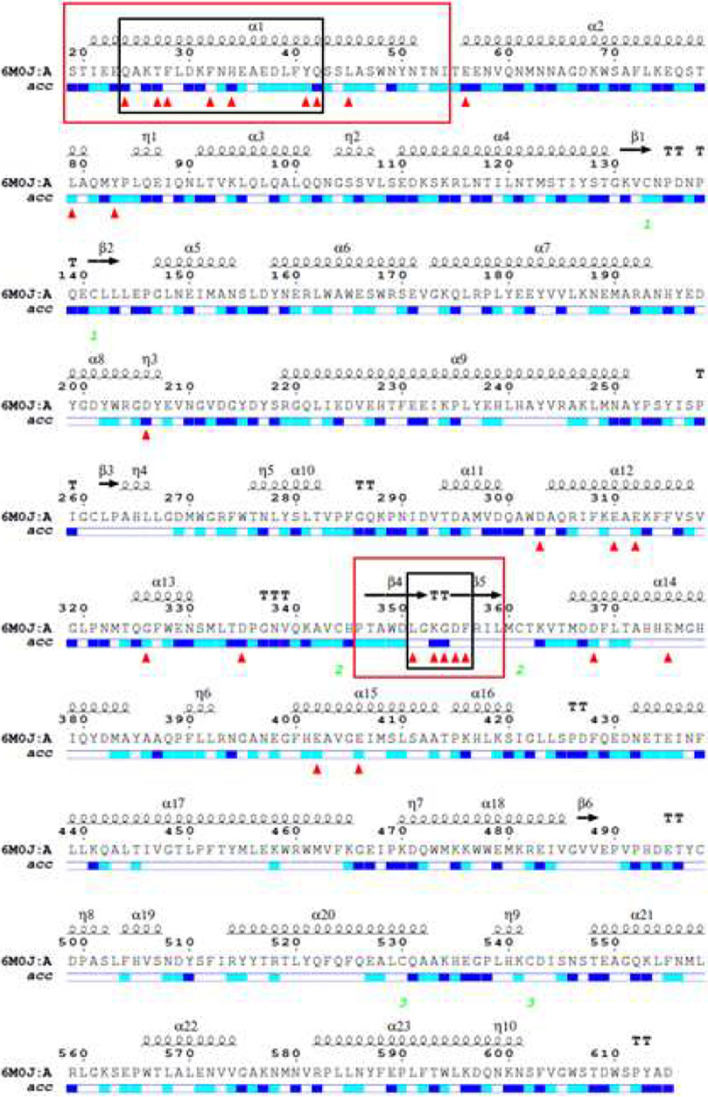
Using ESPript3, the amino acid sequences of ACE2 of 6M0J were aligned with the secondary structure of ACE2, demonstrating the amino acids’ solvent accessibility.. Two regions of ACE2, α1 helical protease domain (PD) and β4 - β5 sheets (red rectangles surrounding the 49 amino acids), contained the majority of the amino acids that interacted with RBD (red triangles)

The interacting energies between RBD and ACE2 for the three protein structures (6M0J, 7C8D, and 7A95) were calculated using MM/GBSA. Table 1 demonstrates the interacting energies between RBD and ACE2 for the three protein structures, showing the highest interacting energy between RBD and ACE2 at − 60.56 kcal/mol. Interface residues between RBD and ACE2 were calculated using MM/GBSA for the three structures, and the repeated residues of ACE2 were aligned with the secondary structure of ACE2 using ESPript version 3, where the interface residues are highlighted in red triangles as illustrated in Fig. 1. The interacting amino acids (black rectangles) were contained inside two regions of ACE2: α1 helical protease domain (PD) and β4 - β5 sheets (red rectangles). The proposed peptide (49 amino acids) was then designed, utilizing the amino acids of the entire length of α1 helix and β4 - β5 sheets inside the red rectangles.

**Table 1:** Using MM/GBSA (kcal/mol). the interacting energies between receptor-binding domain (RBD) and angiotensin-converting enzyme 2 (ACE2) for the three structures (6M0J, 7C8D, and 7A95) were calculated. Bold red highlights the area with the highest interaction energy

Structure	MM/GBSA Scores (kcal/mol)			Average score
6M0J	−60.91	−59.7	−61.06	**−60.56 ± 0.75**
7C8D	− 55.17	−50.37	− 52.92	− 52.82 ± 2.40
7A95	−56.36	− 55.14	− 55.14	−55.55 ± 0.70

The peptide had an estimated molecular weight of 5713.23 kDa and a theoretical isoelectric point (pI) of 4.50, which is less than seven, indicating that the protein has a high proportion of negatively charged residues versus positively charged residues. The instability index (Ii) was determined to be 49.67, indicating solution instability. The hydropathicity index (GRAVY) large average was − 0.710, indicating that it was a hydrophilic protein that could interact in aqueous solutions. The peptide’s aliphatic index (Ai) was 63.88, indicating that it can be stable over a wide temperature range. Taking into account that the estimated half-life of mammalian reticulocytes in vitro was 1.9 hours, while yeast had a half-life of 20 hours, and *E. coli* had a half-life of 10 hours. The extinction coefficient (EC) was calculated to be 13,940 M^− 1^ cm^− 1^, indicating good water solubility and supporting a quantitative study of protein-ligand and protein-protein interaction in solution. Table 2 displays the results of the candidate peptide’s physicochemical properties.

**Table 2:** The physicochemical properties of the peptide. The extinction coefficient (EC) of 13,940 M^− 1^ cm^− 1^ indicates good water solubility and supports a quantitative study of protein-ligand and protein-protein interaction in solution

Peptide Sequence	LENGTH	PI	GRAVY	MW (Da)	Solubility	Half-life (h)	Ii	Ai	EC (M^**−1**^ cm^**− 1**^)
STIEEQAKTFLDKFNHEAEDLFYQSSLASWNYNTNPTAWDLGKGDFRIL	49	4.50	−0.710	5713.23	Good water solubility	1.9	49.67	63.88	13,940

**Fig. 2:**
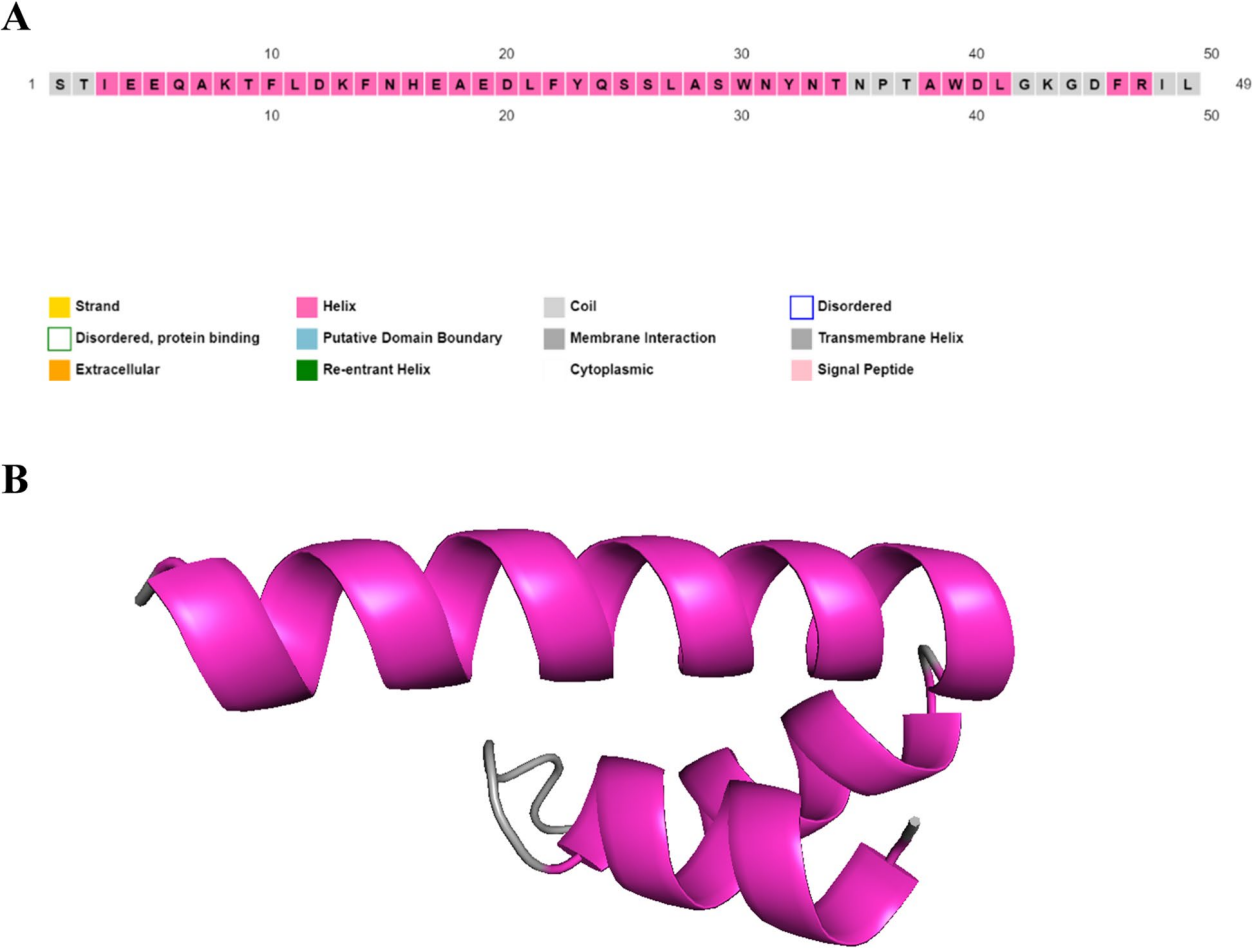
The final peptide’s projected secondary structure. **A** PSIPRED predicted secondary structure. **B** PEP-FOLD3 predicted three-dimensional structure; the helix and coil are, respectively, shown in light magenta and gray

The PSIPRED-predicted secondary structure of the designed peptide is illustrated in Fig. 2A. Using the PEP-FOLD3 web tool, five models of the peptide’s tertiary structure were created and ranked based on the free energies, the ERRAT scores, and the PROVE pass test. The best model was then chosen and designated as the SARS-CoV-2 PEP 49 model, as displayed in Fig. 2B.

**Fig. 3:**
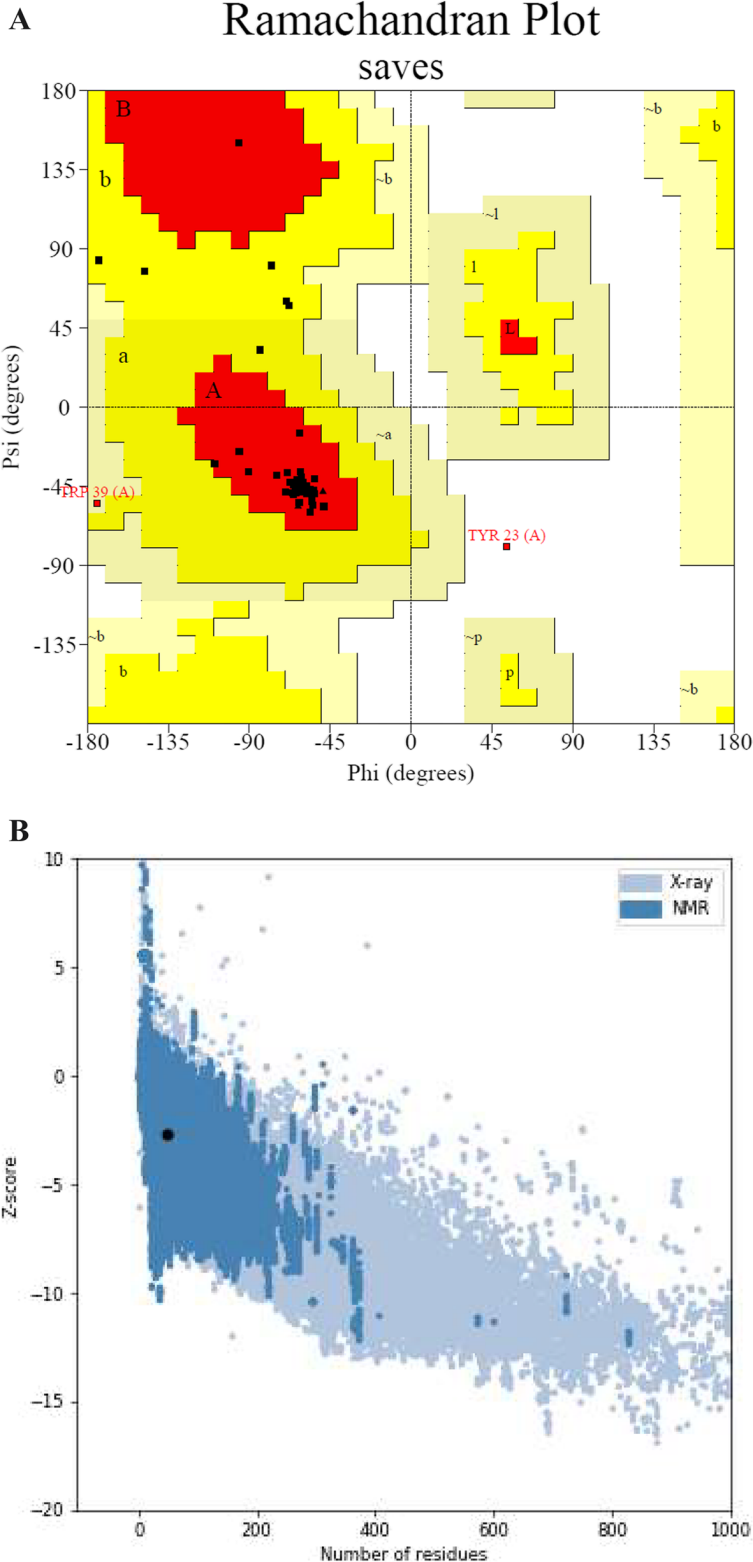
Validation of PEP 49 model’s tertiary structure. **A** More than 97% of the amino acids in the final peptide are in the permitted regions as seen in the Ramachandran plot. **B** ProSA-web plot of the peptide, which results in a Z-score of − 2.68

The energy of the stable conformation of psi (ψ) and phi (Φ) twisting or dihedral angles for each amino acid is determined by the Ramachandran plot. The results of the Ramachandran plot analysis’s tertiary structure validation show that the total percentage of favored and allowed region residues was greater than 97% as displayed in Fig. 3A. The ProSA-web tool was used to validate the quality and potential errors in the crude 3D model, which resulted in a Z-score of − 2.68 for the peptide model as shown in Fig. 3B. The Ramachandran plot and the ProSA-web score both validated the PEP 49 model’s quality.

**Fig. 4:**
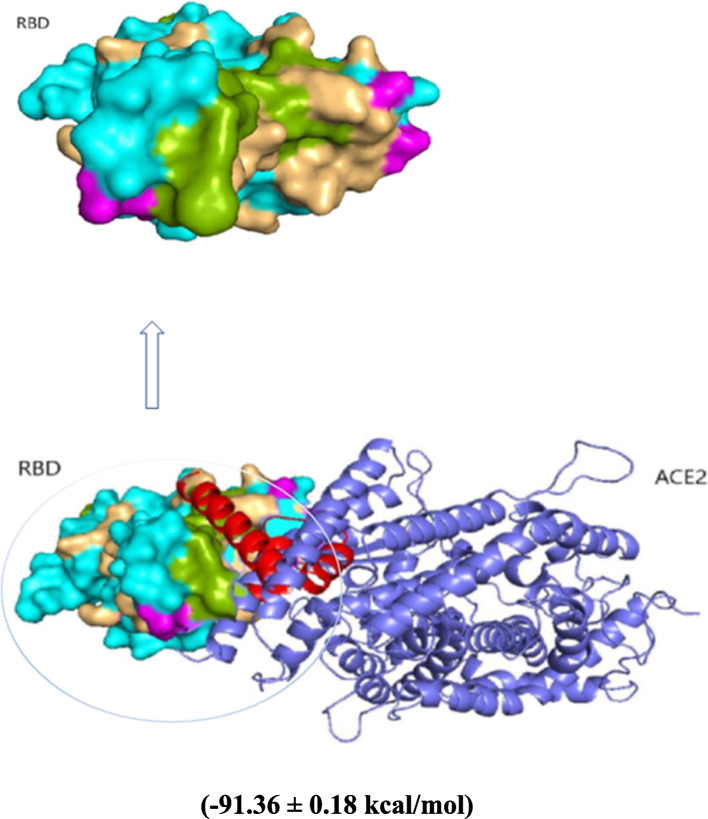
Complexes of PEP 49 (in red) with RBD and ACE2 have a value of (− 91.36 ± 0.18 kcal/mol). The unique interacting residues of PEP 49 are depicted in light green, while the unique interacting residues of ACE2 are depicted in magenta. The common interface residues of PEP 49 model 5; ACE2 and RBD were displayed in light orange

The PEP 49 model was docked to the RBD of the Spike protein 6M0J using ClusPro 2.0. The MM/GBSA interaction energy between the PEP 49 model and RBD was − 91.49 ± 0.18 kcal/mol. The resulting interacting energy was greater than the highest interacting energy (− 60.56 kcal/mol) between RBD and ACE2. Figure 4 displays the common interface residues of the PEP 49 model, ACE2, and RBD in light orange, whereas the unique interacting residues of PEP 49 are shown in light green, and the unique interaction residues of ACE2 are displayed in magenta. As listed in Table 3, the interactions between the RBD and PEP 49 model were investigated using PDBePISA, which included hydrogen bonds and salt bridges. The PEP 49 model produced eight hydrogen bonds and six salt bridges with RBD. This result explains the interaction energy that MM/GBSA measured.

**Table 3:** Utilizing PDBePISA, the interactions between the RBD and PEP 49 peptide models were analyzed. With RBD, the PEP 49 model generated eight hydrogen bonds and six salt bridges

Peptide model-RBD complex					
Hydrogen bonds			Salt bridges		
RBD residue	ACE2 residue	Distance (A^o^)	RBD residue	ACE2 residue	Distance (A^o^)
ARG 346	GLU 4	1.79	ARG 346	GLU 4	3.98
TYR 449	GLU 4	2.04	ARG 346	GLU 4	2.77
GLU 484	LYS 13	1.70	LYS 444	GLU 4	2.64
GLU 484	LYS 13	1.75	LYS 444	GLU 4	2.54
PHE 486	GLU 17	2.39	GLU 484	LYS 13	2.64
ASN 487	GLU 17	2.00	GLU 484	LYS 13	2.54
GLN 493	GLY 42	1.99			
TYR 505	ARG 47	2.38			

## Discussion

In silico molecular docking analysis and simulations targeting the Spike protein revealed insights that designed inhibitors can bind directly at the ACE2 binding site of the Spike protein [54, 55]. However, to have improved affinity, inhibitors need to have complementary conformations that match their target. An inhibitor for the essential amino acids should have selective binding and a low root-mean-square deviation (RMSD) [56–59]. Antiviral peptides can prevent SARS-CoV-2 membrane fusion and could be employed to prevent and treat infections in the future. The use of blocking peptides to target the SARS-CoV-2 Spike protein and fusion cores opens up a new avenue for therapeutic development. Peptide inhibitors are a potential strategy for treating coronavirus infections, according to in silico investigations on SARS-CoV-2 targeting the fusion sites [60–63].

**Fig. 5:**
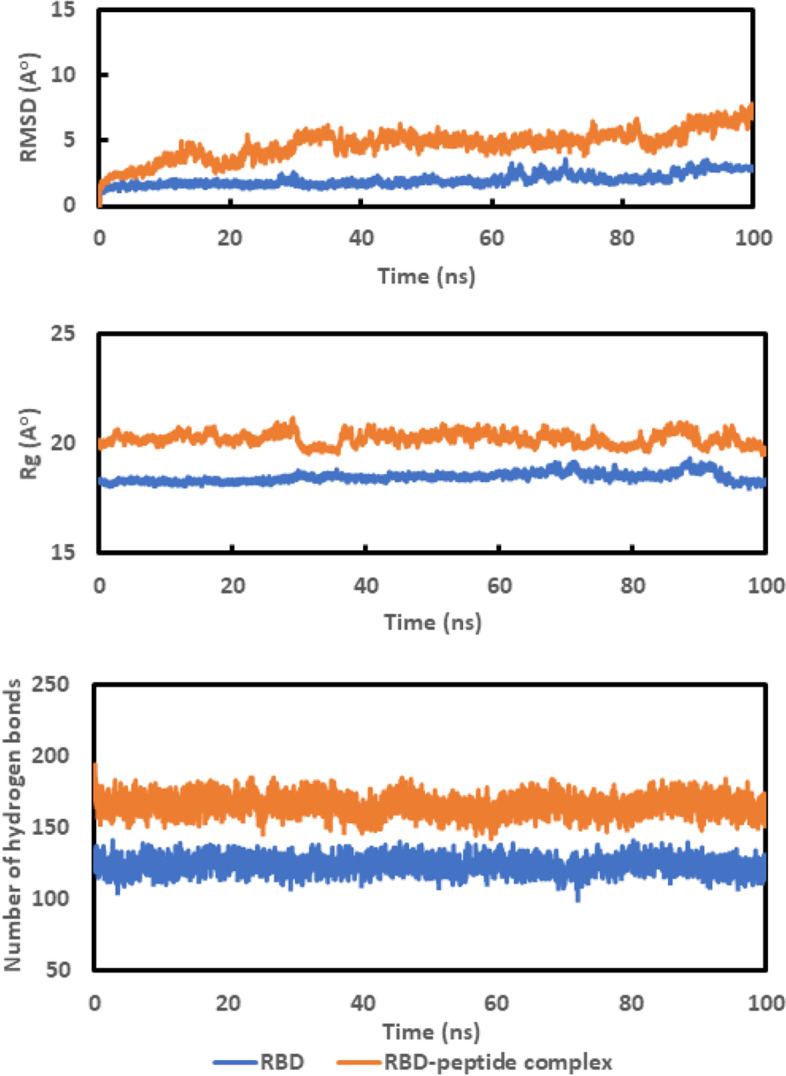
Graph showing the Root Mean Square Deviation (RMSD) for the backbone atoms, the radius of gyration (Rg), the number of hydrogen bonds in the RBD (blue), and the RBD-PEP 49 model 5 complex (orange) throughout the course of a 100 ns simulation

**Fig. 6:**
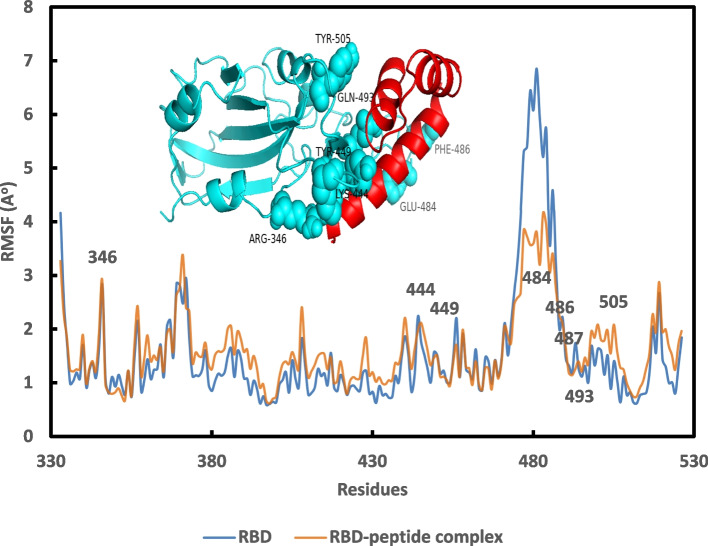
Graph of the average RMSF per residue for the RBD (blue) and RBD-PEP 49 model 5 complex (orange) over 100 ns simulations

The interactions between the ligands and the protein are instantaneous through the docking process, and the interaction may be unstable [64]. The molecular dynamics simulations provided useful information on the stability of the complexes’ molecular interactions. The PEP 49 model had the highest chance of binding to the RBD in this research study. The stability of the complex was assessed using the RMSD for the backbone atoms of RBD and RBD-PEP 49 model complexes in comparison to the starting structures [65]. The RMSD values are plotted in Fig. 5 of RBD (blue) and the RBD – PEP 49 model complex (orange). RBD was stabilized between 5 and 60 nm and RMSD increased after that, while the RBD-PEP 49 model complex was stabilized between 30 and 70 nm and increased after that, which may have returned to the fluctuation of RBD itself. Further, the complex’s stability was assessed by graphing the Radius of gyration (Rg) as a function of time [65]. The calculated Rg values over the simulation time scale are graphed in Fig. 6, where the parameter is stable for the RBD (blue) and the RBD-peptide complex (orange) over the simulation time. The number of hydrogen bonds in the RBD and RBD-peptide complex were shown in Fig. 5 indicating that the number of hydrogen bonds in the complex is greater than the RBD and is approximately stable over the simulation time.

As already observed in Fig. 6, the average RMSF over 100 ns per residue for RBD (blue) and the RBD-peptide complex (orange), indicated a distinct decrease of the RMSF of the residues between 473 and 493 of RBD in the complex with the peptide compared with the free RBD. The presence of residues 484, 486, 487, and 493 in this region, which together form 2 salt bridges and 5 hydrogen bonds with the PEP 49 model, underlines the great stability of these interactions. According to the findings, the SARS-CoV-2 PEP 49 model binds to the ACE2 binding region of RBD with a high affinity, indicating that it might be employed as a possible SARS-CoV-2 Spike protein inhibitor.

## Conclusion

Developing SARS-CoV-2 Spike protein inhibitors has received a lot of attention The designed peptide model created from 49 interface amino acids of ACE2 (SARS-CoV-2 PEP 49) demonstrated a higher binding affinity to SARS-CoV-2 Spike protein than ACE2, implying that it may be used to inhibit SARS-CoV-2. In order to prevent the transmission of the virus, the results of this research could be used to develop therapeutic inhibitors. The designed peptide model could also be used to test new COVID-19 treatment options and their efficacy in experimental research.

## Data Availability

All data generated or analyzed during this study are included in this published article.

## Abbreviations

229E: A species of Alphacoronavirus which infects humans and bats
ACE2: Angiotensin-converting enzyme 2
ClusPro 2.0: Web server for protein-protein docking
COVID-19: 2019 Novel coronavirus
ERRAT scores: “Overall quality factor” for non-bonded atomic interactions measured by a program for verifying protein structures determined by crystallography
HCoVs: Human coronaviruses
HKU1: Species of coronavirus in humans and animals
MERS: Middle East Respiratory Syndrome Coronavirus
MM/GBSA: Poisson–Boltzmann or generalized Born and surface area continuum solvation method
NL63: A species of Alphacoronavirus, specifically a Setracovirus from among the Alphacoronavirus genus
OC43: A member of the species Betacoronavirus 1, which infects humans and cattle
PDB IDs: 6M0J: Crystal structure of SARS-CoV-2 spike receptor-binding domain bound with ACE2
PDB IDs: 7C8D: Cryo-EM structure of cat ACE2 and SARS-CoV-2 RBD
PDBePISA: An interactive web tool for the exploration of macromolecular interfaces
PEP-FOLD3 web tool: A web tool to create the 3D structures of the peptide amino acids
ProSA-web: An interactive web service for the recognition of errors in three-dimensional structures of proteins
PROVE: PROtein Volume Evaluation program
RBD: Receptor-binding domain
RBD-PEP 49: RBD and PEP 49 model complex
Rg: Radius of gyration
RMSF: Root mean square fluctuation
SARS: severe acute respiratory syndrome
SARS-CoV-2: Severe acute respiratory syndrome 2
SARS-CoV-2 PEP 49: Designed model of amino acids with the α1 helix and the β4 - β5 sheets of ACE2
